# Ancestry‐based antihypertensive therapy: Beware of pitfalls

**DOI:** 10.1111/aogs.70272

**Published:** 2026-05-29

**Authors:** Meryam Sugulle, Patji Alnæs‐Katjavivi

**Affiliations:** ^1^ Division of Obstetrics and Gynaecology, Department of Obstetrics Oslo University Hospital Oslo Norway; ^2^ Faculty of Medicine University of Oslo Oslo Norway

Categorization is an important tool in medicine. Successful management of disease depends largely on healthcare professionals' ability to correctly identify and categorize the respective health issue and its signs and symptoms as well as the individual risk factors of the person that is seeking care.

With the aim of equity in health care, medical research has during the past two decades had an increased focus on ethnic disparities in health and disease, as evidenced by a dramatic rise in research output. Savage and Panofsky have pointed out that healthcare personnel, albeit aware of the difficulties related to “race/ethnicity categories,” might have concerns about not taking the variable “race/ethnicity” into account and thus potentially not provide the targeted, person‐centered high‐level care they wish to give.^1^

Whether categorization by skin color, self‐reported ethnic group, or “race” is part of an everyday clinical assessment largely depends on guideline recommendations, if we disregard the issue of implicit bias in healthcare professionals.^2^, ^3^

Despite the valid evidence of dissenting opinion,^4^, ^5^ US and UK management guidelines for the treatment of chronic hypertension in men and non‐pregnant women recommend targeted prescription of first‐line antihypertensives by ethnicity, that is, Black African or African‐Caribbean family origin.^6^, ^7^ For treatment of hypertension in pregnancy, we eagerly await the final results of the Giant PANDA randomized controlled trial.^8^ The trial's main aim was to compare whether initiation of antihypertensive treatment with nifedipine (calcium channel blocker) compared with labetalol (mixed alpha/beta blocker) in women with hypertensive disorders in pregnancy (HDP) reduced development of severe maternal hypertension without increasing adverse perinatal outcome.^8^ A late breaking abstract was presented at the Society for Maternal‐Fetal Medicine Pregnancy Meeting 2026.^9^ The published results suggest a reassuring equivalence in outcome when treating hypertension with either labetalol or modified‐release nifedipine.^9^ Clinical outcomes for 2208 randomized women were similar, irrespective which of the two antihypertensives was initiated: severe hypertension occurring in 12% of those treated with modified‐release nifedipine, compared with 11% of those treated with labetalol; with 8% of participants in each treatment arm experienced severe maternal morbidity, and no adverse maternal events. There were no statistically significant differences in perinatal outcomes.^9^

Seen from a Nordic perspective, the equivalence in maternal and neonatal outcomes is reassuring, with the Danish, Norwegian, Finnish, and Swedish Societies' of Obstetrics and Gynaecology Guidelines recommending labetalol and nifedipine as equal first‐line therapy agents.^10^

However, the authors also report the findings from Giant PANDA showing a statistically significant difference of severe hypertension occurrence among black women (n = not reported) when treated with labetalol (15%) compared to those treated with nifedipine (12%).^9^ Despite the stated intention that trial subgroup analyses would be confined to hypothesis generation,^8^ this finding is included in the abstract's results and conclusion. The abstract's concluding remark is that the results of the trial “should directly inform international guidelines to provide greater options for women with pregnancy hypertension” and seems to apply both for the main result of equivalence of labetalol and nifedipine as equal first‐line therapy agents and the differential findings in black women.^9^

Aware of the fact that a late breaking abstract, albeit published in an indexed obstetric journal, does not correspond to a peer‐reviewed original research article, we argue that here lies the difficulty in this presentation of (preliminary) subgroup analyses results which again leads us to raise several questions.

How should the Nordic, other European countries, or indeed Africa, with different “Black” populations^11^ than the UK translate the trial's results? Direct adaption into guidelines could potentially do harm by categorizing groups of pregnant women based on a skin color that is assumed to be uniformly indicative of shared ethnic ancestry and thus globally valid. Currently Nordic countries, in keeping with other national guidelines for the management of HDP, make no recommendations regarding race‐based prescribing of antihypertensives.^10^ However, the Swedish Society of Obstetrics and Gyneacology Guideline remarks that “women with African ethnic background are less receptive for beta‐blockers.”^12^

In clinical practice, whether “Black” or “non‐Black”: a pregnant woman with a hypertensive disorder is more than likely to respond adequately to both labetalol and nifedipine, given that overall 88% of all included women and 85% in the “Black” group had the effect of labetalol in the Giant PANDA study.^9^ Introducing bias might also pose risks for the “non‐Black” non‐responders that are numerically more frequent, for example, in the Nordic setting.

Where do the assumptions of the effect of skin color as a proxy for ancestry on receptiveness for certain antihypertensive agents come from? And even more important: Are there any indications that these assumptions are biologically plausible?

In their comprehensive review on ethnic disparities in hypertensive disorders of pregnancy, Conti‐Ramsden et al. point out that to date, adequately powered genome‐wide association studies for preeclampsia are lacking.^13^ The authors underline that the most recent multinational biobank study in its discovery phase only included 20 women with and 1406 without preeclampsia/eclampsia of African ancestry from one US biobank of a total of 17 150 cases and 451 241 controls.^14^ Furthermore, Conti‐Ramsden et al.^13^ refer to awaited study results concerning APOLI1 genetic variants as contributors to preeclampsia in women of West African ancestry, at the time of study protocol publication 384 mother–child pairs constituted the preeclampsia group of a total of 773 mother–child pairs included in the study.^15^ Of note, the West African study participants represent 5 different ethnic groups plus a 6th group denominated “others,”^15^ a reminder that persons originating from the African continent show greater difference in genetic variation than persons from any other continent in the world.^11^ Therefore, there is currently no sufficient evidence linking genetic variants and thus biological causality and preeclampsia.

Concerning antihypertensive agents, Conti‐Ramsden et al.^13^ stress that contemporary national and international HDP guidelines do not make any recommendations on targeted prescription of antihypertensive agents based on ethnicity or “race,” but that such recommendations are to be found in UK and US guidelines for management of chronic hypertension outside pregnancy.^6^, ^7^ The authors inform the reader about the assumption underlying these recommendations, namely that there supposedly exists a low‐renin hypertension phenotype which is more prevalent in persons with African ancestry or “Black ethnic backgrounds.”^13^ This hypertension phenotype would biologically translate to a decreased receptiveness for beta‐blockers and angiotensin‐converting enzyme inhibitors and an increased sensitivity for calcium channel blockers.^13^ Conti‐Ramsden et al. summarize that three small studies of chronic hypertension in pregnancy, with methodological limitations (*n* = 117, 23 and 120 women of Black African/Caribbean ethnic background), demonstrated respectively lower renin levels and a lower proportion of responders to labetalol.^13^ Conti‐Ramsden et al. call for caution regarding the use of ethnicity as a proxy for “biological” risk and make the point, that the influence of non‐pregnancy guidelines recommending ethnicity/“race”‐based antihypertensive management might influence clinicians dealing with obstetric patients.^13^

In conclusion, while acknowledging the global research effort aiming at reducing inequity in general and in women's health specifically, there are several pitfalls we need to be aware of when translating research findings into international or national valid clinical guidelines. Set aside the past and current lack of proof of biological causality between skin color/ethnicity/ancestry/“race” and preeclampsia or antihypertensive treatment responsiveness:

The main clinical pitfall is that black skin color is taken for globally valid shared ancestry, and this might lead to misunderstood adaptations of recommendations despite the lack of external validity as exemplified by the Swedish guidelines.

Encouraged by the comparable effect of initiating nifedipine versus labetalol for the treatment of hypertension in pregnancy, we eagerly await the full publication of the Giant PANDA study, calling out to treat the findings of the subgroup analysis with caution.

## AUTHOR CONTRIBUTIONS

**Meryam Sugulle:** Conceptualization; writing – original draft; writing – review and editing. **Patji Alnæs‐Katjavivi:** Conceptualization; writing – original draft; writing – review and editing.

## CONFLICT OF INTEREST STATEMENT

None declared.

## Data Availability

Data sharing not applicable to this article as no datasets were generated or analysed during the current study.
